# Uncommon Thromboembolic Events in Young Adults: A Rare Cause Uncovered

**DOI:** 10.7759/cureus.76962

**Published:** 2025-01-05

**Authors:** Mohamed El Hacen Mohamed Yile, Elodie Berteau, Nabil Ramdani, Thomas Mouyen, Nadine Ferrier

**Affiliations:** 1 Department of Cardiology, Mohammed VI University Hospital Center, Abdelmalek Esaadi University, Tangier, MAR; 2 Cardiology, Hospital Center Jacques Lacarin, Vichy, FRA

**Keywords:** b12 dysfunction, extensive thrombosis, nitrous oxide, pulmonary embolism, recreational nitrous oxide use

## Abstract

Nitrous oxide (N₂O), widely used as a medical anesthetic, has increasingly been misused recreationally, especially among young people, due to its accessibility and perceived safety. However, prolonged use and high doses can cause severe complications, particularly through functional vitamin B12 dysfunction. These complications include neurological impairments, hematologic abnormalities, and thromboembolic events, such as pulmonary embolism. We report the case of a 20-year-old male with a history of chronic nitrous oxide consumption who presented with extensive bilateral lower limb thrombosis, pulmonary embolism, and peripheral neuropathy. Imaging confirmed extensive iliofemoral thrombosis and bilateral pulmonary embolism. The patient was treated with anticoagulation, vitamin B12 supplementation, and other supportive measures. This case highlights the need to raise awareness about the risks of recreational nitrous oxide use and underscores the importance of timely intervention to prevent severe complications.

## Introduction

Nitrous oxide (N₂O), widely used as a medical anesthetic with analgesic and anxiolytic properties, is an odorless and colorless gas that has increasingly been misused for recreational purposes, particularly among young people [1]. Despite the common misconception regarding its safety, numerous reports have highlighted its harmful effects on the neurologic, hematologic, and hemodynamic systems, particularly with prolonged use and high doses. These effects are largely due to functional vitamin B12 deficiency, which leads to hyperhomocysteinemia, a condition responsible for a hypercoagulable state that increases the risk of vascular thrombosis [2]. We report the case of a 20-year-old male with a history of chronic nitrous oxide consumption who presented with extensive bilateral lower limb thrombosis and bilateral pulmonary embolism. This case highlights the unusual presentation in our patient, who lacked both hematologic and neurologic complications while manifesting a rapid thromboembolic event. It underscores the importance of raising awareness about the potential complications of long-term nitrous oxide use, a substance often mistakenly perceived as safe.

## Case presentation

We present the case of a 20-year-old male with a three-year history of nitrous oxide consumption (two to three small canisters per week), cigarette smoking (10 cigarettes/day), and moderate alcohol use, likely linked to a recent emotional trauma as reported by the patient. He denied using any other recreational drugs or having a family history suggestive of familial thrombophilia. The patient presented to the emergency department with a three-day history of diffuse pain and swelling in his left lower limb. On admission, he was hemodynamically stable, with regular heart sounds, no murmurs, and clear vesicular breath sounds. The ECG revealed sinus rhythm with a normal axis, no conduction or repolarization abnormalities, and no signs of ischemia. Laboratory tests showed elevated CRP at 172 mg/L, leukocytosis at 11 G/L, and significantly elevated D-dimers at 13,567 ng/mL (normal <500 ng/mL). Renal and hepatic functions were preserved, with no troponin or N-terminal pro-B-type natriuretic peptide (NT-proBNP) elevation. Vitamin B12 levels were within the normal range (220 pmol/L; normal 145-569 pmol/L), and the thrombophilia workup, including protein C, protein S, and antithrombin III levels, was normal.

**Figure 1:**
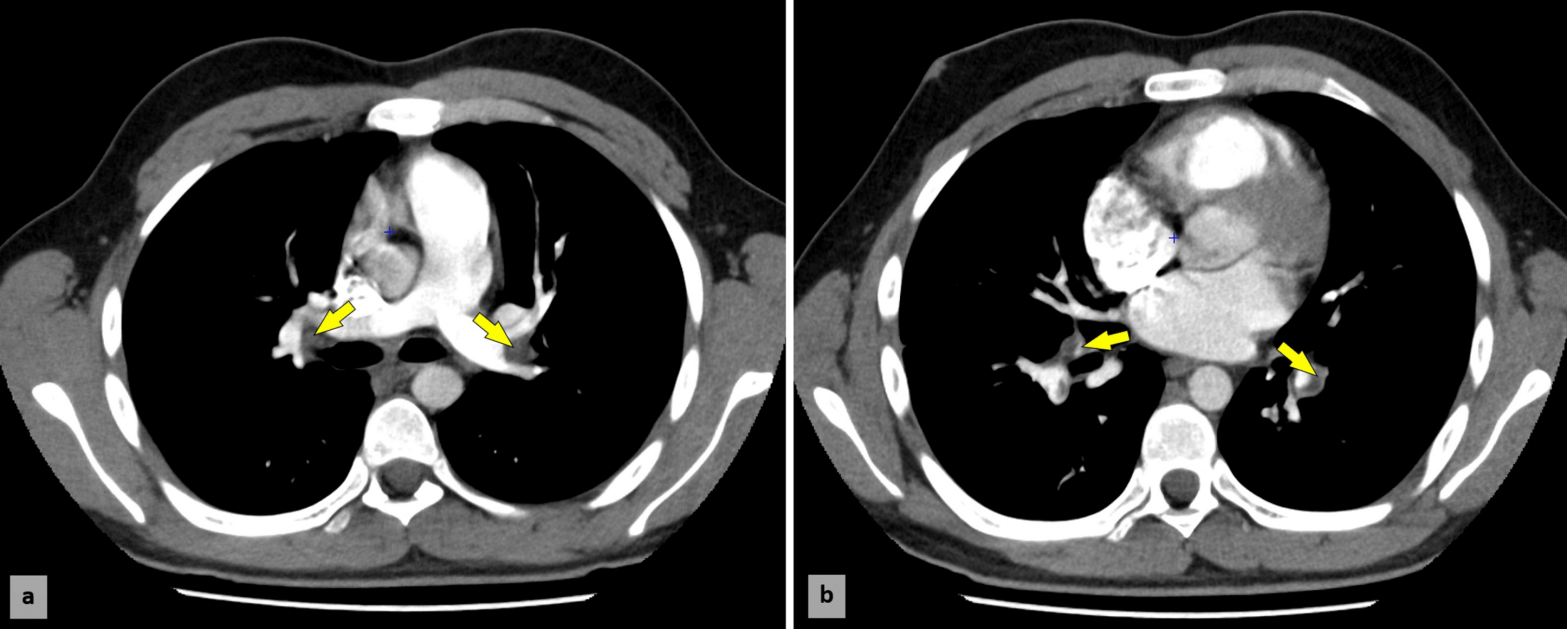
Post-contrast axial CT scan of the thorax reveals bilateral pulmonary emboli (arrows)

CT angiography revealed bilateral pulmonary embolism at the lobar level and extensive left iliofemoral thrombosis (Figure 1), while echocardiography showed preserved left ventricular function, right heart chamber dilation without inversion of the right ventricular (RV)/left ventricular (LV) ratio, and a pulmonary artery systolic pressure of 23 mmHg. Doppler ultrasound confirmed deep venous thrombosis in the right posterior tibial and fibular veins, and extensive thrombosis in the left lower limb involving the common iliac vein, femoral veins, popliteal vein, and distal veins, with compression at the termination of the left common iliac vein due to May-Thurner syndrome. Although the patient reported some episodes of paresthesia over the past few weeks, both the neurologic examination and the electroneuromyogram did not reveal any abnormal neurologic function.

The patient was diagnosed with a low-risk pulmonary embolism and initiated on anticoagulation therapy with apixaban (20 mg/day for seven days, followed by 5 mg/day). Thrombectomy with angioplasty was also considered for the extensive left-sided thrombosis. During hospitalization, the patient showed significant clinical improvement and was discharged with a treatment plan including amoxicillin 1 g/day for three days, paracetamol 500 mg as needed, cyanocobalamin (vitamin B12) 1 mg/day, and continuation of apixaban. At a two-month follow-up, after complete cessation of nitrous oxide use, the patient’s symptoms had resolved, with imaging confirming full resolution of the pulmonary embolism and lower limb thrombosis.

## Discussion

Recreational nitrous oxide (N₂O), also known as “laughing gas,” is an increasingly prevalent phenomenon among young people due to its accessibility and relatively fewer side effects. It is becoming increasingly prevalent in some countries, such as the UK, Netherlands, and Australia. Primarily used as an anesthetic agent [3], nitrous oxide is appealing because of its easy availability, perceived harmlessness, and brief duration of effects. It is often obtained through nitrous oxide whippets used for whipped cream [4]. However, nitrous oxide causes functional vitamin B12 deficiency by irreversibly inactivating the vitamin, leading to complications such as macrocytic anemia and vitamin B12-related peripheral neuropathy [5].

A range of symptoms is associated with nitrous oxide use, especially with long-term consumption or high doses. These include central and peripheral neurologic complications, thromboembolic events, and hematologic issues, most notably macrocytic anemia [5]. Neurologic symptoms are the most frequently observed, affecting nearly 96% of patients, predominantly due to elevated homocysteine levels, which interfere with myelin production [6]. These symptoms usually include confusion, hallucinations, weakness, and imbalance [1]. Thrombotic events are less common, with only a few cases of pulmonary embolism described in the literature [7]. Venous circulation is primarily affected due to its low flow; however, rare cases of arterial thrombosis have also been reported [4]. Other reported complications include myocardial infarction, pneumomediastinum, and cases of death linked to hypoxia [8].

In our case, the pulmonary embolism was directly associated with extensive lower limb thrombosis, likely exacerbated by immobilization, as the patient reported prolonged bed rest following the onset of a generalized left limb swelling. Immobilization is a well-known risk factor for thromboembolic complications [9]. Elevated homocysteine levels, caused by vitamin B12 inactivation from nitrous oxide use, may further increase the risk of thromboembolic events. Homocysteine elevation disrupts endothelial function, platelet activity, and fibrinolysis, all of which can contribute to thrombosis [5].

Discontinuing nitrous oxide use, supplementing vitamin B12, and initiating targeted rehabilitation are critical for restoring normal neurologic function. Thromboembolic complications require anticoagulant therapy, such as therapeutic-dose heparin or direct oral anticoagulants, until the thrombosis resolves [6]. The management of cytopenia varies depending on its severity and the affected cell lineages. In our case, transfusions were unnecessary since the patient remained hemodynamically stable with normal blood counts. Neurologic function often improves over weeks to months with high-dose vitamin B12 supplementation, although complete remission of neurologic symptoms is rarely achieved [10].

## Conclusions

Nitrous oxide, an anesthetic agent widely known among young people for its euphoric effects and relatively low side effects, has been linked to severe complications, particularly with long-term and high-dose use. This case underscores the importance of considering the recreational use of nitrous oxide as a potential cause of serious thromboembolic complications, even in otherwise healthy young individuals. A prompt and effective therapeutic strategy should address both the management of thromboembolic events and the underlying causes.

## References

[1] Nguyen N, Cao J, Carlson D, Kong L, Diaz G (2024). Nitrous oxide use precipitates pulmonary embolism: a case report. Cureus.

[2] Cellai AP, Lami D, Antonucci E (2014). Hyperhomocysteinemia in patients with pulmonary embolism is associated with impaired plasma fibrinolytic capacity. J Thromb Thrombolysis.

[3] Emmanouil DE, Quock RM (2007). Advances in understanding the actions of nitrous oxide. Anesth Prog.

[4] den Uil SH, Vermeulen EG, Metz R, Rijbroek A, de Vries M (2018). Aortic arch thrombus caused by nitrous oxide abuse. J Vasc Surg Cases Innov Tech.

[5] Pedersen OB, Hvas AM, Grove EL (2021). A 19-year-old man with a history of recreational inhalation of nitrous oxide with severe peripheral neuropathy and central pulmonary embolism. Am J Case Rep.

[6] Parein G, Bollens B (2023). Nitrous oxide-induced polyneuropathy, pancytopenia and pulmonary embolism: a case report. J Med Case Rep.

[7] Oulkadi S, Peters B, Vliegen AS (2022). Thromboembolic complications of recreational nitrous oxide (ab)use: a systematic review. J Thromb Thrombolysis.

[8] Chanarin I (1982). The effects of nitrous oxide on cobalamins, folates, and on related events. Crit Rev Toxicol.

[9] Wells PS, Anderson DR, Rodger M (2000). Derivation of a simple clinical model to categorize patients probability of pulmonary embolism: increasing the models utility with the SimpliRED D-dimer. Thromb Haemost.

[10] Massey TH, Pickersgill TT, J Peall K (2016). Nitrous oxide misuse and vitamin B12 deficiency. BMJ Case Rep.

